# Modulation of growth hormone receptor-insulin-like growth factor 1 axis by dietary protein in young ruminants

**DOI:** 10.1017/S0007114519003040

**Published:** 2020-03-28

**Authors:** Caroline S. Firmenich, Nadine Schnepel, Kathrin Hansen, Marion Schmicke, Alexandra S. Muscher-Banse

**Affiliations:** 1 Institute for Physiology and Cell Biology, University of Veterinary Medicine Hannover, 30173 Hannover, Germany; 2 Clinic for Cattle, Endocrinology Laboratory, University of Veterinary Medicine Hannover, 30173 Hannover, Germany

**Keywords:** Growth hormone receptor, Insulin-like growth factor 1 synthesis, Insulin, Reduced-protein diet, Ruminants

## Abstract

A reduced protein intake causes a decrease in insulin-like growth factor 1 (IGF1) concentrations and modulates Ca homoeostasis in young goats. IGF1 is synthesised by the liver in response to stimulation by growth hormone (GH). Due to rumino-hepatic circulation of urea, ruminants are suitable for investigating the effects of protein reduction despite sufficient energy intake. The present study aimed to investigate the impact of a protein-reduced diet on the expression of components of the somatotropic axis. Male young goats were divided into two feeding groups receiving either a control diet (20 % crude protein (CP)) or a reduced-protein diet (9 % CP). Blood concentrations of IGF1 and GH were measured, and a 24-h GH secretion profile was compiled. Moreover, ionised Ca and insulin concentrations as well as mRNA and protein expression levels of hepatic proteins involved in GH signalling were quantified. Due to the protein-reduced diet, concentrations of ionised Ca, insulin and IGF1 decreased significantly, whereas GH concentrations remained unchanged. Expression levels of the hepatic GH receptor (GHR) decreased during protein reduction. GHR expression was down-regulated due to diminished insulin concentrations as both parameters were positively correlated. Insulin itself might be reduced due to reduced blood Ca levels that are involved in insulin release. The protein-reduced diet had an impact on the expression of components of the somatotropic axis as a disruption of the GH–IGF1 axis brought about by diminished GHR expression was shown in response to a protein-reduced diet.

In intensive livestock farming, feeding ruminants a dietary protein content close to their demand (11–12 % crude protein^(1)^) is preferable for economic and environmental reasons. Due to efficient rumino-hepatic circulation of urea^(2)^, ruminants are thought to easily cope with a reduced protein and therefore diminished N intake as long as the energy supply is maintained^(3)^.

However, it was shown that decreased dietary protein intake caused massive changes in mineral homoeostasis and vitamin D metabolism in young goats^(4^^,^^5)^; this being reflected by reduced levels of blood Ca, 1,25-dihydroxyvitamin D_3_ (calcitriol) and insulin-like growth factor 1 (IGF1). Protein restriction was also shown to have an impact on Ca homoeostasis, IGF1 and vitamin D metabolism in monogastric species like humans and rats^(6^^–^^8)^.

Calcitriol is synthesised by the action of mitochondrial enzyme 1*α*-hydroxylase (CYP27B1) in the kidney^(9)^. IGF1 has a direct stimulating effect on the synthesis of calcitriol by modulating the expression and activity of renal CYP27B1^(10)^. A positive correlation between IGF1 concentration and CYP27B1 expression was shown, underlining the impact of IGF1 on CYP27B1 synthesis and therefore circulating levels of calcitriol^(11)^.

Therefore, it was assumed that IGF1 was the link between reduced dietary protein intake and diminished levels of calcitriol and blood Ca in the present animals^(12)^. IGF1 is synthesised by the liver after pulsatile secretion of growth hormone (GH) from the pituitary gland. The synthesis and secretion of GH are regulated by the hypothalamus, stimulated by GH-releasing hormone and inhibited by somatostatin^(13)^. GH mediates its action by binding to the membranous hepatic GH receptor (GHR) dimer and therefore initiates the Janus kinase-signal transducers and activators of transcription (JAK-STAT) pathway, leading to IGF1 secretion among others^(14)^. Through activating JAK2, GH is known to activate STAT 1, 3 and 5^(15)^. Suppressor of cytokine signalling 1 (SOCS1) was shown to inhibit the intrinsic activity of JAK2, SOCS2 was shown to bind the phosphorylated GHR, whereas SOCS3 binds JAK2 and the phosphorylated GHR^(16)^, hence exerting a negative feedback on the JAK-STAT pathway^(16)^. The JAK-STAT signalling pathway is controlled via negative feedback by SOCS proteins^(16)^. Besides the JAK-STAT pathway, signal transduction via tyrosine-protein kinase src (Src) activating extracellular signal-regulated kinases (ERK1/2) is involved in mediating the intracellular effect of GH^(14)^. In blood, circulating IGF1 is bound to IGF1 binding proteins (IGFBP) which serve as carrier proteins and regulate IGF1 turnover, transport and distribution^(17)^. This binary complex binds to the acid-labile subunit (ALS), leading to the formation of a ternary complex that plays an important role in the biology of circulation IGF1^(18)^.

The hypothesis of the present study was that the somatotropic axis is disrupted during a reduced-protein diet in young goats, resulting in decreased IGF1 concentrations and unchanged GH levels. Therefore, the present study aims to compile a molecular characterisation of components of the somatotropic axis. Due to diminished levels of IGF1 occurring during a reduced dietary protein intake, 24-h GH secretion patterns were examined. Based on the great similarity in sequences between IGF1 and insulin and the ability of both proteins to bind to the IGF1 receptor and to the hepatic insulin receptor (INSR), concentrations of insulin were measured. Finally, the expression of fibroblast growth factor 21 (FGF21), a hepatokine, was quantified because FGF21 is an endocrine signal of protein restriction in monogastric species^(19)^.

## Materials and methods

### Animals and feeding regimen

The protocols of the animal feeding and handling experiments were approved by the Animal Welfare Commissioner of the University of Veterinary Medicine Hannover (Hannover, Germany) and were in line with the German Animal Welfare Law.

Male, coloured, German goats (*Capra aegagrus hircus*) with an initial weight of 15·4 (sem 0·51) kg were divided into two feeding groups with one group receiving a diet with elevated protein levels (20 % crude protein; *n* 8 animals) and the second group with reduced protein levels (9 % crude protein; *n* 9 animals) for about 6 weeks. Animals of the same feeding regimen were housed together in groups of four or five animals with water available *ad libitum*.

The individual feeding of each goat was performed at 07.00 and 16.00 hours daily. Each animal was brought to the stable alley in front of its trough for individual feed intake. The straw was fed 30 min before the pelleted concentrates. To calculate the intake of nutrients and minerals for each animal, all offered and refused feeds were monitored. Each animal was fed 55 g pelleted concentrate per kg metabolic body size (kg body weight (BW)^0·75^) and 14 g/kg BW^0·75^ of chopped wheat straw divided into two equal meals per d.

Animals were weighed weekly. The feed content of DM, crude ash, crude fibre, crude fat and crude protein was determined by means of the Weende analysis (proximate analysis), the standard procedure of the German Association of Agricultural Analytic and Research Institutes (Verband Deutscher Landwirtschaftlicher Untersuchungs- und Forschungsanstalten). The amounts of acid-detergent fibre and neutral-detergent fibre were measured by a method described by Van Soest *et al.*^(20)^. The two diets were almost isoenergetic, containing approximately 12 MJ metabolisable energy/kg DM. The components and composition of the diets are shown in Table 1. To adjust the weight of the protein-reduced diet, Sipernat 22S, a fine particle silica, which cannot be metabolised and which is commonly used as a digestibility marker due to its inert structure^(21)^, was added.

**Table 1.** Components and composition of wheat straw and pelleted concentrate diets*

	Wheat straw	Control	Reduced protein
Components (as-fed basis) (g/kg)			
Soyabean meal	–	120	77
Urea	–	30	–
Wheat starch	–	376	366
Beet pulp	–	418	400
Molasses	–	–	10
Soyabean oil	–	10	31
Mineral–vitamin mix†	–	10	10
MgHPO_4_·3H_2_O	–	9·5	10·5
NaH_2_PO_4_·2H_2_O	–	7	7
CaCO_3_	–	19·5	19·5
Sipernat 22S‡	–	–	69
Composition			
DM (g/kg)	912	911	900
Nutrients (g/kg DM)			
Crude ash	65·8	70·3	123·3
CP	3	20	9
Acid-detergent fibre	541·7	80·1	76·7
Neutral-detergent fibre	823·5	152·6	144·4
Crude fat	8·8	23·1	40·0
Urea	BDL	32·8	6·1
Ca	2·2	12·3	11·9
P	0·7	5·0	4·8
Vitamin D_3_ (IU/kg)	<1000	<1000	<1000
ME (MJ/kg DM)	6·7	13·3	12·1

CP, crude protein; BDL, below detection level; ME, metabolisable energy.

* Composition expressed as fed (analysed by the Association of German Agricultural Investigation and Research Center).

† Mineral–vitamin mix per kg: 12·1 g Ca; 1·9 g Na; 2·2 g Mg; 400 mg (1 200 000 IU) vitamin A; 0·3 mg (12 000 IU) vitamin D_3_; 10 g vitamin E; 6335 mg Zn; 3000 mg Mn; 201 mg Co; 201 mg iodine; 15 mg Se.

‡ Sipernat type 22S (Evonik Industries AG) is a fine particle silica with high oil absorption capacity. It is widely used as a flow regulator, anti-caking and dusting agent especially in the food and feed industry.

### 24-h growth hormone and insulin-like growth factor 1 profile

To compile a 24-h profile of GH and IGF1 secretion patterns, blood samples were taken throughout 24 h at intervals of 20 min from each animal in each group. Animals were implanted with permanent catheters in the jugular vein, and at each time point, 1 ml venous blood was taken with EDTA-coated syringes (Sarstedt AG & Co. KG) to obtain plasma samples. As one goat could not be implanted with a catheter, it was excluded from the sampling.

Blood was separated by centrifugation (2000 ***g*** at room temperature, 15 min). Additionally, at three time points (11.00, 19.00 and 03.00 hours), blood samples were collected with serum syringes (Sarstedt AG & Co. KG) to obtain serum for measuring concentrations of IGF1. The plasma and serum samples were stored at −20°C for subsequent analysis.

Plasma GH and serum IGF1 concentrations were analysed in the Clinic for Cattle, Endocrinology Laboratory, University of Veterinary Medicine, Hannover, using in-house enzyme-linked immunosorbent assay or by RIA (Beckman Coulter).

### Blood sampling and biochemical determinations of blood parameters

Blood samples were always collected at the same time in the morning before slaughtering to avoid circadian effects by puncturing the vena jugularis with EDTA-coated, lithium heparin-coated syringes and serum syringes (Sarstedt AG & Co. KG). Blood was separated by centrifugation (see above). Plasma and serum samples were stored at −20°C.

Plasma concentrations of urea were measured using a commercial kit (R-Biopharm AG). Ionised Ca and glucose concentrations were measured in whole blood samples. For the determination of ionised Ca, an ion-sensitive electrode (Chiron Vaccines & Diagnostics GmbH) was used. Glucose levels were detected via the method of mutant Q-GDH-based blood glucose monitor using an Accu-Chek Performa glucose metre (Roche Diagnostic GmbH). Plasma concentrations of insulin were measured by ELISA, and triiodothyronine (T3) concentrations were analysed by competitive chemiluminescence immunoassay in the Clinic for Cattle, Endocrinology Laboratory, University of Veterinary Medicine, Hannover. Plasma concentrations of total protein were detected using a bromocresol green albumin assay kit (Sigma-Aldrich Chemie GmbH). Serum concentrations of TAG were measured in the Clinic for Cattle, Chemical and Clinical Laboratory, University of Veterinary Medicine, Hannover. Protein expressions of IGFBP2, IGFBP3, IGFBP4 and IGFBP5 in plasma were analysed commercially by quantitative Western ligand blot analysis as previously described (Ligandis)^(22)^. The measurements of serum IGF1 and plasma GH concentrations in samples were taken before slaughtering as previously explained.

### Hepatic tissue sampling and histological slices

At the end of the experimental feeding after 6 weeks, the goats were slaughtered after captive bolt stunning by exsanguination. To avoid circadian effects, slaughtering was always performed at the same time in the morning. For technical reasons, four goats per d were killed from an alternating group. On one day, five goats were killed.

Liver samples were removed within 5 min post-mortem and immediately rinsed with ice-cold saline (0·9 % NaCl), frozen in liquid N_2_ and stored at −80°C until further preparation. To assess the texture of the hepatic tissue, histological slices were made and dyed with haematoxylin–eosin as previously described using a standard procedure^(23)^. Moreover, Sudan stains were made by the Department of Pathology, University of Veterinary Medicine, Hannover to determine the level of fat in liver tissue as previously described^(23)^.

### Gene expression analysis

Total RNA was isolated using the RNeasy plus Mini Kit (Qiagen) with genomic DNA eliminator spin columns in accordance with the manufacturer’s protocol. The RNA concentrations were measured by UV-visible spectrophotometry (Thermo Fisher Scientific GmbH, NanoDrop One). To verify the quality of the isolated RNA, the RNA integrity number was evaluated with an RNA 6000 nanoassay for an Agilent 2100 Bioanalyzer (Agilent Technologies GmbH).

Using random hexamers, oligo-dT primers and TaqMan Reverse-Transcription Reagents (Applied Biosystems Deutschland GmbH), 200 ng isolated hepatic RNA was reverse-transcribed in accordance with the manufacturer’s protocol. The primers used for the production of recombinant DNA were derived either from caprine, ovine or bovine sequences. Primers were designed to span exon-exon junctions. The GHR1A primer pair was designed to amplify the most common variance of the hepatic GHR^(24)^. Primer sequences, exon spanning region, amplicon length and efficiency are summarised in Table 2. Expressions of hepatic ALS, ERK2, FGF21, GHR1A, IGF1, IGFBP2, IGFBP3, INSR, JAK2, RPS9, SOCS1, SOCS2, SOCS3 and STAT5B were determined using SYBR Green PCR assays. Reaction mixtures (20 µl) contained KAPA SYBR FAST Universal Master Mix (PEQLAB Biotechnology GmbH), 200 nm specific primers and 16 ng reverse-transcribed complementary DNA (cDNA). PCR products were amplified (95°C, 3 min; forty cycles of 95°C, 10 s and 60°C, 30 s). Primer sequences, exon spanning region, amplicon length and efficiency are summarised in Table 3. For quantifying the expressions of hepatic 18S rRNA, *β*-actin and GAPDH mRNA, primers and probes were purchased from TIB MOLBIOL. Reaction mixtures (20 µl) contained TaqMan Universal PCR Master Mix (Applied Biosystems), 300 nm specific primers, 100 nm specific probe and 16 ng cDNA. The PCR product was amplified (50°C, 2 min; 95°C, 10 min; forty cycles of 95°C, 15 s and 60°C, 1 min) and analysed using a real-time PCR cycler (CFX96^TM^; Bio-Rad Laboratories GmbH). Absolute copy numbers were determined using calibration curves generated with cloned PCR fragment standards^(26)^. Specificity of the amplicons was verified by sequencing (Seqlab Microsynth GmbH) and using NCBI Blast (http://blast.ncbi.nlm.nih.gov/Blast.cgi). Expressions of genes of interest were normalised to a quotient of 18S rRNA/*β*-actin as two constantly expressed housekeeping genes in liver tissue during the applied dietary treatment which was determined by using NormFinder software (https://moma.dk/normfinder-software). Reactions were performed in duplicate and included no template water controls.

**Table 2.** Primers and probes used for TaqMan assays

Gene	Sense and antisense primers (5′ → 3′), probes	Exon location	Amplicon length (bp)	Amplification efficiency (%)	Accession number	Source
18S rRNA	AAAAATAACAATACAGGACTCTTTCG	–	127	85	KY129860	(11)
	GCTATTGGAGCTGGAATTACCG					
	FAM-TGGAATGAGTCCACTTTAAATCCTTCCGC-BBQ					
*β*-Actin	CTCAGAGCAAGAGAGGCATCC	3	111	99	XM_018039831.1	(25)
	GCAGCTCGTTGTAGAAGGTGTG					
	FAM-CAAGTACCCCATTGAGCACGGC-TMR					
GAPDH	CAAGGTCATCCATGACCACTTT	7–8	95	100	NM_001190390	(26)
	CGGAAGGGCCATCCACA					
	FAM-CTGTCCACGCCATCACTGCCACCC-TMR					

18S rRNA, 18S ribosomal RNA; GAPDH, glyceraldehyde 3-phosphate dehydrogenase.

**Table 3.** Primers used for SYBR Green assays

Gene	Sense and antisense primers (5′ → 3′)	Exon location	Amplicon length (bp)	Amplification efficiency (%)	Accession number	Source
ALS	TACCTGGACCACAACCTCGT	2	202	86	XM_018040889.1	(27)
	AGTGCAGGTCCTTGAAGGTG					
ERK2	TCCCGAATGCTGACTCCAAA	–	185	86	JQ308787.1	Present study
	CTTGGGCAAGTCATCCAAATACA					
FGF21	CCTCTACACGGATGATGCCC	1–3	216	100	XM_005692688.3	Present study
	GCTTTGGGGTCAAAGTGCAG					
GHR1A	CGTCTCTGCTGGTGAAAACA	4, 5	203	101	NM_176608.1	(22)
	GGATGTCGGCATGAATCTCT					
IGF1	ATCAGCAGTCTTCCAACCCAAT	1, 2	192	105	XM_005680531.3	Present study
	ACACACGAACTGGAGAGCATCC					
IGF2	TGGCCCTACTGGAGACCTAC	3, 4	101	104	NM_001287041.1	Present study
	CACGGGGTATGCTGTGAAGT					
IGFBP2	GTCCTGGAACGGATCTCCAC	2, 3	121	100	NM_001314299.1	Present study
	TCTTGCACTGTTTGAGGTTGT					
IGFBP3	GCATGAGACAGAATACGGGC	2–4	183	106	NM_001314219.1	Present study
	TCCACACACCAGCAGAAACC					
INSR	TATGGGACTGGGGCAAACAC	6, 7	177	101	XM_018051134.1	Present study
	TTTCACAGGATGCCTGGTCC					
JAK2	GTGCTGGCCGGCATAATCTA	20–22	188	101	XM_018052022.1	Present study
	ATTCCTTGTCGCCAGATCCC					
RPS9	CGCCTCGACCAAGAGCTGAAG	2, 3	65	73	XM_018063497.1	(28)
	CTCCAGACCTCACGTTTGTTCC					
SOCS1	CTCGTACCTCCTACCTCTTCATGTT	2	95	99	XM_864316.5	(28)
	CACAGCAGAAAAATAAAGCCAGAGA					
SOCS2	CTTTGAAACAGCTGCGTCCC	3	163	95	XM_018047627.1	Present study
	AGCAGGCATGTATCCCCCTA					
SOCS3	AAGACCTTCAGCTCCAAGAGCG	1	113	110	XM_004013097.3	Present study
	AGCAGCAAGTTCGCTTCGC					
Src	GGCTTCTACATCACCTCCCG	8, 9	131	90	XM_018057837.1 to	Present study
	CCTTGAGTCTGCGGCTTAGA					
					XM_018057840.1	
STAT1	TCTCTACCCGTCGTGGTGAT	16, 17	159	89	XM_018061547.1	Present study
	CCAGCTCAGCACCTCTGAAA					
STAT3	GACCTGGAGACGCACTCATT	14–16	156	93	NM_001314278.1	Present study
	CACTTGATCCCACGTTCCGA					
STAT5B	GCCACAGATCAAGCAAGTGG	16–18	247	104	XM_018065118.1	Present study
	GGGGATCCACTGACTGTCCAT				XM_018065117.1	

ALS, acid-labile subunit; ERK2, extracellular-signal regulated-kinase 2; FGF21, fibroblast growth factor 21; GHR1A, growth hormone receptor 1A; IGF1, insulin-like growth factor 1; IGF2, insulin-like growth factor 2; IGFBP2, insulin-like growth factor binding protein 2; IGFBP3, insulin-like growth factor binding protein 3; INSR, insulin receptor; JAK2, Janus kinase 2; RPS9, ribosomal protein S9; SOCS1, suppressor of cytokine signalling 1; SOCS2, suppressor of cytokine signalling 2; SOCS3, suppressor of cytokine signalling 3; Src, tyrosine-protein kinase src; STAT1, signal transducers and activators of transcription 1; STAT3, signal transducers and activators of transcription 3; STAT5B, signal transducers and activators of transcription 5B.

### Tissue protein extraction and Western blot analysis

Hepatic cytosol was isolated as described elsewhere^(26)^. Hepatic crude membranes were prepared using a FastPrep-24 High Speed Homogenizer (MP Biomedicals GmbH). Briefly, frozen hepatic tissue was agitated with zirconia balls in a 20 mmol/l TRIS buffer containing 250 mmol/l sucrose, 5 mmol/l EGTA and 5 mmol/l MgSO_4_.7H_2_O at pH 7·5. Afterwards, phosphatase inhibitor cocktail tablets (cOmplete ULTRA, Roche) and protease inhibitor cocktail tablets (PhosSTOP, Roche) were added in accordance with the manufacturer’s instructions on the day of extraction, followed by centrifugation (2000 ***g*** at 4°C, 20 min). In the following step, the homogenate was centrifuged (40 000 ***g*** at 4°C, 60 min). The supernatant was removed, and the pellet was dissolved in a pH 7·4 buffer containing 10 mmol/l TRIS and 150 mmol/l NaCl. Protein concentrations were measured with a commercial protein assay using Bradford reagent (Serva Electrophoresis GmbH). For pERK1/2, ERK1/2, JAK2, SOCS2, Src, STAT1, STAT3 and STAT5B, 20 µg hepatic cytosol and for GHR and INSR, 20 µg hepatic crude membranes were separated by 8·1 % SDS-PAGE and transferred onto nitrocellulose membranes (GE Healthcare Europe GmbH) using the Trans-Blot Turbo transfer system (Bio-Rad). The primary antibodies used were chosen due to their specificity for the particular caprine protein or species cross-reactivity for caprine or bovine target protein. Moreover, protein sequences were verified for their homology using NCBI Blast (http://blast.ncbi.nlm.nih.gov/Blast.cgi). To ensure that no non-specific signal was detected, membranes were incubated only with the secondary antibody. For pERK1/2, ERK1/2, JAK2, SOCS2, INSR, Src, STAT1 and STAT3, membranes were blocked in TRIS-buffered saline containing 0·1 % Tween 20 and for GHR and STAT5B, membranes were blocked in PBS containing 0·1 % Tween 20 with 5 % fat-free milk powder, respectively. For pERK1/2 and ERK1/2, the membranes were incubated overnight at 4°C with anti-pERK1/2 and ERK1/2 antibody (Cell Signaling Technology Europe B.V.). After detecting pERK1/2 antibody, membranes were treated with a pH 2·0 stripping buffer as described elsewhere^(29)^ and used again for ERK1/2 antibody detection. For detecting caprine GHR protein, two different antibodies were used: a commercial antibody (Santa Cruz Inc.) and a self-made antibody (Davids Biotechnology GmbH). The membranes were incubated overnight at 4°C with the commercial or with the self-made anti-GHR antibody. The self-made antibody was successfully pre-incubated with the corresponding antigenic peptide (data not shown). For detecting JAK2, SOCS2, Src, STAT1, STAT3, STAT5B and INSR protein, membranes were incubated overnight at 4°C with anti-JAK2 antibody (Cell Signaling), anti-SOCS2 antibody (Cell Signaling), anti-Src antibody (Cell Signaling), anti-STAT1 antibody (Cell Signaling), anti-STAT3 antibody (Cell Signaling), anti-STAT5B antibody (GeneTex Inc.) or with anti-INSR antibody (Santa Cruz), respectively. After washing, membranes were incubated with corresponding secondary anti-rabbit or anti-mouse horseradish peroxidase-conjugated antibody. The proteins were detected with a chemiluminescence system and the ChemiDoc MP (Bio-Rad). Densitometric measurements were performed using Image Lab 5.2.1 software (Bio-Rad). For semi-quantifying the proteins, values of the investigated proteins were normalised to the amount of applied total protein amounts per lane.

### Statistical analysis

Sample size (minimum *n* 7/group) was determined based on metabolic data from previous work^(5)^ with a statistical power of 0·8 and *α* error of 0·05. Data are given as means with their standard errors if not stated otherwise and number of animals (*n*). All data were normally distributed. Comparisons of values between the two feeding groups were analysed by unpaired Student’s *t* test. To test for linear relationship between two variables, a simple correlation analysis with Pearson’s correlation coefficient was calculated. All statistical analyses were performed using GraphPad Prism version 7.04 (GraphPad Software; www.graphPad.com). *P* < 0·05 was set to be significant, and *P* < 0·1 was used to define trends.

## Results

### Intake, body weight and daily weight gain during reduced protein feeding

All animals were clinically healthy during the present study. Mean daily intakes of DM, concentrate, N and Ca were calculated for each animal individually. Feed efficiency was calculated as the difference between the final and initial weight divided by the calculated feed intake during this period. DM, concentrate and Ca intake did not differ between the two groups. Feed efficiency was not different, while N intake was significantly reduced in the animals receiving the protein-reduced diet (Table 4). Daily energy supply remained within the range recommended by the Society of Nutrition Physiology (GfE) for young ruminating goat kids. The initial BW (control 15·3 (SEM 0·67) kg, reduced protein 15·6 (SEM 0·80) kg; *P* = 0·74) and the final BW of the goats were not different due to any dietary intervention (control 23·6 (SEM 0·38) kg, reduced protein 22·7 (SEM 1·09) kg; *P* = 0·47). Daily weight gain during the period of experimental feeding was not affected by any diet and ranged from 0·14 to 0·16 (SEM 0·008) kg/d.

**Table 4.** Mean daily intake of DM, concentrate, nitrogen, calcium and feed efficiency of growing goats receiving a protein-reduced diet (Mean values with their standard errors)

Items	Control (*n* 8)		Reduced protein (*n* 9)		*P*
	Mean	sem	Mean	sem	
DM intake (g/d)	567	6·87	536	22·7	0·18
Concentrate intake (g/d)	419	8·32	406	21·2	0·58
Feed efficiency (kg/kg)	0·79	0·02	0·76	0·02	0·26
N intake (g/d)	14·5	0·23	6·2	0·30	<0·001
Ca intake (g/d)	5·15	0·10	5·05	0·26	0·74

### 24-h growth hormone and insulin-like growth factor 1 profile during reduced protein feeding

Concentrations of IGF1 were significantly diminished at all time points (11.00, 19.00 and 03.00 hours) due to the protein-reduced feeding (Table 5). The number of 24-h GH pulses blood sampling did not differ significantly between the two feeding groups. Moreover, total GH secretion during the time of blood sampling showed no significant differences due to any feeding regimen (Table 6).

**Table 5.** Effects of a reduced-protein diet on insulin-like growth factor 1 concentrations (ng/ml) in young goats (Mean values with their standard errors)

Parameters	Control (*n* 8)		Reduced protein (*n* 8)		*P*
	Mean	sem	Mean	sem	
11.00 hours	404	36·8	257	23·6	0·005
19.00 hours	397	32·1	279	27·0	0·014
03.00 hours	408	29·8	299	22·3	0·011

**Table 6.** Effects of a reduced-protein diet on growth hormone (GH) pulsatility given in number of pulses per 24 h and GH secretion given as total GH in 24 h in young goats (Mean values with their standard errors)

Parameters	Control (*n* 8)		Reduced protein (*n* 8)		*P*
	Mean	sem	Mean	sem	
Number of pulses < 10	51·88	7·23	54·38	7·9	0·59
Number of pulses ≥ 10	20·13	7·23	26·5	7·87	0·59
Total hormone secretion (ng/ml per 24 h)	589·18	92·68	687·75	108·53	0·50

### Blood parameters during reduced protein feeding

**Fig. 1.**
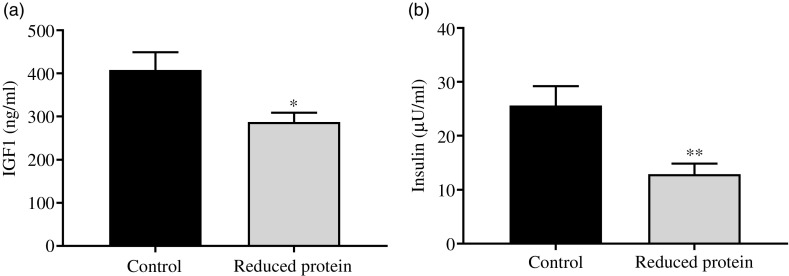
Concentration of insulin-like growth factor 1 (IGF1) (a) and insulin (b) in plasma of goats receiving a protein-reduced diet. Data are expressed as mean values with their standard errors; control = eight animals and reduced protein = nine animals. * *P* < 0·05, ** *P* < 0·01 *v.* control diet.

**Fig. 2.**
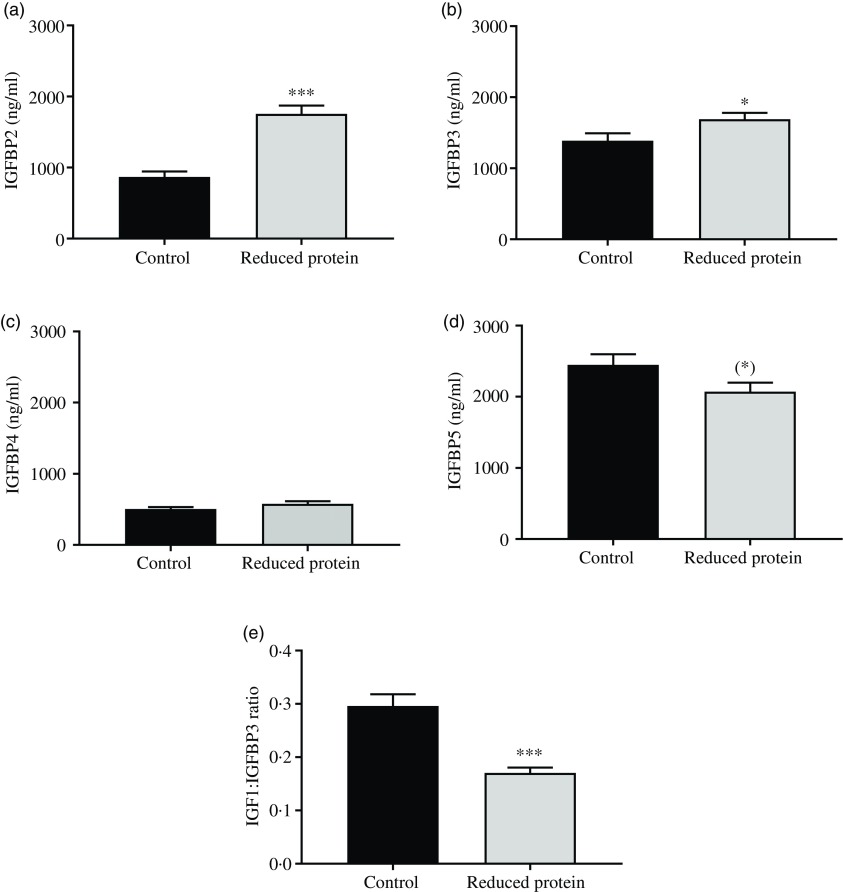
Concentration of insulin-like growth factor 1 (IGF1) binding protein 2 (IGFBP2) (a), of IGFPB3 (b), of IGFBP4 (c), IGFPB5 (d) and of free IGF1 as a quotient of IGF1 and IGFBP3 (e) in plasma of goats receiving a protein-reduced diet. Data are expressed as mean values with their standard errors; control = eight animals and reduced protein = nine animals. (*) *P* < 0·10, * P < 0·05, *** *P* < 0·001 *v.* control diet.

The subsequent sections present the results of the different blood parameters for the two feeding regimens on the day of slaughter. Results are described in detail where differences between the groups occurred (Table 7). Plasma urea, concentrations of blood ionised Ca, serum IGF1 (Fig. 1(a)) and plasma insulin (Fig. 1(b)) concentrations decreased significantly when dietary protein was reduced. A significant positive correlation between blood ionised Ca and plasma insulin concentration was determined (*r* 0·55, *P* = 0·02) and between plasma insulin and urea concentrations (*r* 0·51, *P* = 0·04). Plasma concentration of IGFBP2 (Fig. 2(a)) and IGFBP3 (Fig. 2(b)) protein increased significantly due to the protein-reduced feeding. Plasma concentration of IGFBP4 protein was not modulated by any dietary intervention (Fig. 2(c)). The plasma IGFBP5 protein concentration decreased by trend due to the protein-reduced feeding (Fig. 2(d)). A negative correlation between IGFBP2 plasma protein concentrations and serum IGF1 concentration was detected (*r* −0·57, *P* = 0·02), whereas a positive correlation between IGFBP5 plasma protein concentration and serum IGF1 concentration was measured (*r* 0·78, *P* = 0·002). No significant correlation was detected between concentrations of IGFBP3 or IGFBP4 protein and serum levels of IGF1. For determining the amount of free IGF1, a quotient between IGF1 and IGFBP3 was calculated which was significantly reduced in the low-protein-fed group (Fig. 2(e), *P* < 0·001).

**Table 7.** Effects of a reduced-protein diet on blood parameters of young goats (Mean values with their standard errors)

Items	Control (*n* 8)		Reduced protein (*n* 9)		*P*
	Mean	sem	Mean	sem	
Total blood					
Ionised Ca (mmol/l)	1·27	0·01	1·20	0·02	0·04
pH	7·44	0·007	7·45	0·006	0·21
Plasma					
Urea (mmol/l)	5·22	0·41	0·86	0·09	<0·0001
Insulin (µU/ml)	25·6	3·6	12·9	1·98	0·006
Glucose (mg/dl)*	76·5	1·45	73·11	1·98	0·20
Total protein (mg/ml)	49·4	1·15	51·3	2·01	0·46
GH (ng/ml)	20·2	2·0	21·3	2·5	0·75
T3 (ng/ml)	2·19	0·12	2·04	0·07	0·27
Serum					
IGF1 (ng/ml)	408	41·0	287	21·8	0·02
TAG (mmol/l)	0·28	0·02	0·28	0·02	0·93

GH, growth hormone; T3, triiodothyronine; IGF1, insulin-like growth factor 1.

* To convert glucose in mg/dl to mmol/l, multiply by 0.0555.

### Assessment of liver tissue

Microscopic evaluations of the histological liver slices dyed with haematoxylin–eosin indicated no differences between the two feeding groups. The fat staining showed only a few small diffuse fatty vacuoles in both groups. Therefore, no accumulation of fat or hepatic damage was assumed (data not shown).

### Expression of acid-labile subunit, extracellular signal-regulated kinases 2, fibroblast growth factor 21, growth hormone receptor 1A, insulin-like growth factor 1, insulin-like growth factor 2, IGF1 binding protein 2, IGF1 binding protein 3, insulin receptor, Janus kinase 2, suppressor of cytokine signalling 1, suppressor of cytokine signalling 2, suppressor of cytokine signalling 3and signal transducers and activators of transcription 5BmRNA during reduced protein feeding

The integrity of the isolated RNA of all hepatic tissue samples, expressed as RNA integrity number, was at least 8·7 (sem 0·04). The mRNA expression levels of ERK2, IGF2, IGFBP3, INSR, JAK2 and STAT5B showed no alternation due to the protein-reduced feeding. Furthermore, the mRNA expression of SOCS1 was not altered, whereas the mRNA expression levels of SOCS2 showed an increase by trend and SOCS3 mRNA expression was significantly enhanced in the protein-reduced- fed animals. Expression levels of FGF21 and IGFBP2 increased significantly due to the low-protein feeding. The protein-reduced diet led to a significant decrease in ALS, IGF1 and GHR1A mRNA expression levels (Table 8). A significant positive correlation between GHR mRNA expression levels and plasma urea concentrations (*r* 0·64, *P* = 0·006), plasma insulin concentration (*r* 0·61, *P* = 0·01) and between GHR mRNA expression and serum IGF1 concentration (*r* 0·64, *P* = 0·006) was detected.

**Table 8.** Relative amounts of hepatic ALS, ERK2, FGF21, GHR1A, IGF1, IGF2, IGFBP2, IGFBP3, INSR, JAK2, SOCS1, SOCS2, SOCS3, Src, STAT1, STAT3 and STAT5B mRNA expression normalised to hepatic quotient of 18S rRNA/*β*-actin in goats fed a protein-reduced diet (Mean values with their standard errors)

Items	Control (*n* 8)		Reduced protein (*n* 9)		*P*
	Mean	sem	Mean	sem	
ALS	1·98 × 10^6^	0·47 × 10^6^	0·68 × 10^6^	0·14 × 10^6^	0·01
ERK2	1·14 × 10^–2^	0·05 × 10^−2^	1·10 × 10^–2^	0·11 × 10^–2^	0·70
FGF21	3·47 × 10^2^	1·07 × 10^2^	41·76 × 10^2^	16·49 × 10^2^	0·04
GHR1A	3·04 × 10^7^	0·59 × 10^7^	1·66 × 10^7^	0·13 × 10^7^	0·03
IGF1	2·49 × 10^6^	0·51 × 10^6^	1·38 × 10^6^	0·20 × 10^6^	0·05
IGF2	3·83 × 10^7^	0·62 × 10^7^	3·03 × 10^7^	0·38 × 10^7^	0·27
IGFBP2	0·13 × 10^6^	0·02 × 10^6^	0·40 × 10^6^	0·07 × 10^6^	0·002
IGFBP3	0·11 × 10^6^	0·01 × 10^6^	0·11 × 10^6^	0·02 × 10^6^	0·73
INSR	0·10 × 10^6^	0·02 × 10^6^	0·11 × 10^6^	0·01 × 10^6^	0·59
JAK2	0·37 × 10^6^	0·07 × 10^6^	0·40 × 10^6^	0·07 × 10^6^	0·75
SOCS1	0·07 × 10^5^	0·01 × 10^5^	0·17 × 10^5^	0·09 × 10^5^	0·36
SOCS2	0·79 × 10^6^	0·16 × 10^6^	1·30 × 10^6^	0·20 × 10^6^	0·08
SOCS3	0·07 × 10^6^	0·02 × 10^6^	0·14 × 10^6^	0·02 × 10^6^	0·04
Src	6·72 × 10^2^	1·14 × 10^2^	9·92 × 10^2^	2·14 × 10^2^	0·21
STAT1	0·17 × 10^5^	0·02 × 10^5^	0·17 × 10^5^	0·04 × 10^5^	0·96
STAT3	0·16 × 10^5^	0·03 × 10^5^	0·17 × 10^5^	0·03 × 10^5^	0·84
STAT5B	1·94 × 10^5^	0·49 × 10^5^	1·59 × 10^5^	0·37 × 10^5^	0·58

ALS, acid-labile subunit; ERK2, extracellular signal-regulated kinase 2; FGF21, fibroblast growth factor 21; GHR1A, growth hormone receptor 1A; IGF1, insulin-like growth factor 1; IGF2, insulin-like growth factor 2, IGFBP2, IGF1 binding-protein 2; IGFBP3, IGF1 binding-protein 3; INSR, insulin receptor; JAK2, Janus kinase 2; SOCS1, suppressor of cytokine signalling 1; SOCS2, suppressor of cytokine signalling 2; SOCS3, suppressor of cytokine signalling 3; Src, tyrosine-protein kinase src; STAT1, signal transducers and activators of transcription 1; STAT3, signal transducers and activators of transcription 3; STAT5B, signal transducers and activators of transcription 5.

### Expression of extracellular signal-regulated kinases 1/2, growth hormone receptor, insulin receptor, Janus kinase 2, suppressor of cytokine signalling 2, tyrosine-protein kinase src, signal transducers and activators of transcription 1, signal transducers and activators of transcription 3and signal transducers and activators of transcription 5Bprotein during reduced protein feeding

**Fig. 3.**
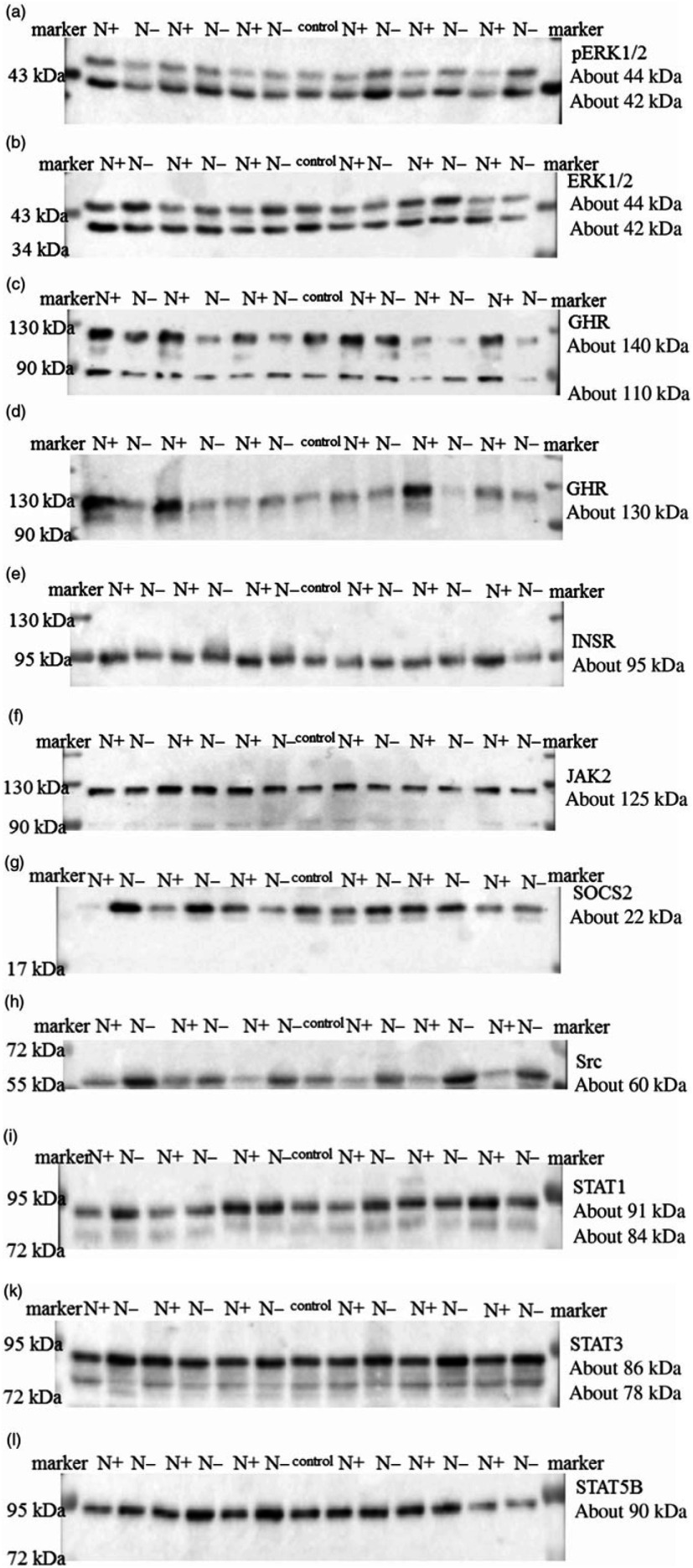
Representative signals of phosphorylated extracellular signal-regulated kinase 1/2 (pERK1/2) (a), extracellular signal-regulated kinase 1/2 (ERK1/2) (b), growth hormone receptor (GHR; Santa Cruz) (c), growth hormone receptor (GHR; Davids Biotechnology) (d), insulin receptor (INSR) (e), Janus kinase 2 (JAK2) (f), suppressor of cytokine signalling 2 (SOCS2) (g), tyrosine-protein kinase src (Src) (h), signal transducers and activators of transcription 1 (STAT1) (i), signal transducers and activators of transcription 3 (STAT3) (k) and signal transducers and activators of transcription 5B (STAT5B) (l) protein in hepatic tissue of goats receiving a protein-reduced diet.

The ERK1/2 and pERK1/2 protein expression levels were detected as two bands of 42 and 44 kDa, respectively. Protein expression levels of EKR1/2 showed no significant differences due to the applied diets. Phosphorylation levels of EKR1/2 were not significantly different between the two feeding groups. Protein expression of GHR was detected as two bands of 110 and 140 kDa in accordance with the manufacturer’s instructions (Santa Cruz Biotechnology) and decreased significantly due to the protein-reduced diet. GHR protein expression was analysed a second time with a self-made antibody (130 kDa band; Davids Biotechnology GmbH). Again, a significant decrease in GHR expression was shown due to the protein-reduced diet. The protein expression of INSR was detected as a band of 95 kDa, SOCS2 protein expression was detected at 22 kDa and Src protein expression was detected as a band of approximately 60 kDa. The protein expression of hepatic INSR, SOCS2 and Src increased significantly due to reduced protein intake. The protein expression of JAK2 was detected at 125 kDa. The protein expression of STAT1 was detected at two bands as 84 and 91 kDa, STAT3 protein expression was detected as two bands of 78 and 86 kDa and STAT5B protein expression was detected at 90 kDa. Protein expression levels of JAK2 and STAT1 did not differ between the two feeding groups. Protein expression levels of STAT3 increased significantly and STAT5B showed an increase by trend due to the protein-reduced diet. Representative Western blots of the investigated proteins are shown in Fig. 3(a)–(l). All data are summarised in Table 9.

**Table 9.** Relative amounts of ERK1/2 and pERK1/2, GHR, INSR, JAK2, SOCS2, Src, STAT1, STAT3 and STAT5B protein expression normalised to total protein amounts in the liver of goats fed a protein-reduced diet (Mean values with their standard errors)

Item	Control (*n* 8)		Reduced protein (*n* 9)		*P*
	Mean	sem	Mean	sem	
ERK1/2	5·07 × 10^–3^	0·43 × 10^–3^	4·97 × 10^–3^	0·47 × 10^–3^	0·88
pERK1/2	1·59 × 10^–3^	0·24 × 10^–3^	2·05 × 10^–3^	0·35 × 10^–3^	0·26
GHR (Santa Cruz)	3·94 × 10^–3^	0·48 × 10^–3^	2·13 × 10^–3^	0·53 × 10^–3^	0·02
GHR (Davids Biotechnology)	3·46 × 10^–3^	0·51 × 10^–3^	1·73 × 10^–3^	0·57 × 10^–3^	0·04
INSR	0·80 × 10^–3^	0·073 × 10^–3^	1·07 × 10^–3^	0·062 × 10^–3^	0·01
JAK2	3·55 × 10^–3^	0·37 × 10^–3^	3·30 × 10^–3^	0·25 × 10^–3^	0·54
SOCS2	1·38 × 10^–3^	0·16 × 10^–3^	2·51 × 10^–3^	0·35 × 10^–3^	0·01
Src	3·68 × 10^–3^	0·75 × 10^–3^	8·14 × 10^–3^	0·73 × 10^–3^	<0·001
STAT1	4·84 × 10^–3^	0·61 × 10^–3^	5·79 × 10^–3^	0·38 × 10^–3^	0·20
STAT3	7·15 × 10^–3^	0·56 × 10^–3^	9·45 × 10^–3^	0·67 × 10^–3^	0·005
STAT5B	1·44 × 10^–3^	0·13 × 10^–3^	1·90 × 10^–3^	0·16 × 10^–3^	0·03

ERK1/2, extracellular signal-regulated kinases; GHR, growth hormone receptor 1A; INSR, insulin receptor; JAK2, Janus kinase 2; SOCS2, suppressor of cytokine signalling 2; Src, tyrosine-protein kinase Src; STAT1, signal transducers and activators of transcription 1; STAT3, signal transducers and activators of transcription 3; STAT5B, signal transducers and activators of transcription 5B.

## Discussion

The aim of the present study was to determine the influence of a protein-reduced diet on components of the somatotropic axis in young goats. It could be shown for the first time that during growth, this feeding regimen had an impact on parts of the somatotropic axis.

The limitation of the present study was the restricted concentrate feeding instead of *ad libitum* feeding. Therefore, a potential protein appetite resulting from a stimulation of feed intake and higher body fat which was observed in monogastric species during protein restriction^(30)^ could not be considered. However, in histological tests with liver tissue, no accumulation of fat during a protein restriction could be observed and plasma TAG concentrations remained unchanged.

The hepatic expression of FGF21 mRNA was elevated directly by protein restriction like in mice and rats^(19)^ rather than by energy restriction shown by same BW gain, final weight and unaffected plasma triiodothyronine concentrations, an energy-dependent indicator which drops due to energy insufficiency^(31)^.

Due to increased IGFBP2 and IGFBP3 protein levels, compensatory mechanisms were presumed to maintain IGF1 availability for the target tissues. In cultured rat hepatocytes, synthesis of IGFBP2, IGFBP3 and IGFBP4 was shown to be affected by insulin and IGF1, whereas a response to GH was not observed^(32)^. Nutritional restriction increased IGFBP2 by an enhanced transcription rate linked to a decreased protein intake^(33)^ as was shown in the present study. As the quotient between IGF1 concentrations and IGFBP3 was significantly reduced due to the low-protein feeding, a decrease in concentrations of free circulating IGF1 was assumed. Unfortunately, immunoassays measuring IGF1 ignore the effects of IGFBPs on the interaction between IGF1 and its receptor^(34)^. Therefore, it would be better to use a method allowing a comparison between serum levels of IGF1 available for its receptor, free IGF1 and total IGF1^(34)^. Reduced ALS mRNA expression in the animals fed the low-protein diet might lead to higher concentrations of IGF1 bound to the binary complex which is able to pass the vascular epithelium and reach the target tissue. The level of ALS is directly dependent on GH and the regulation occurs at the level of transcriptional activation of the ALS gene in the liver^(35)^. In rat liver, it was found that ALS mRNA and nuclear transcripts were reduced in animals made GH deficient by hypophysectomy. Furthermore, it was shown that GH stimulated ALS promotor activity which was mediated by functional GHR at GH concentrations within the physiological range^(35)^. As GH concentration and pulsatility did not differ between the two groups, a disruption of the somatotropic axis resulting in diminished IGF1 concentrations despite constant levels of GH occurred during a reduced-protein diet.

Unaltered IGF2 mRNA levels support this idea, as GH signalling has only little effect on inducing the IGF2 gene in comparison with the IGF1 gene. As both diets fed in the present study were almost isoenergetic, only a lack of N intake could cause reduced GHR expression in the present study, which is affirmed by a positive correlation between urea concentrations and GHR expression levels. Interestingly, there is strong evidence that insulin is essential for GH stimulation of hepatic IGF1 production and that insulin regulates hepatic GHR biosynthesis and surface translocation^(36)^. In human hepatic cell lines, the action of insulin on GHR was measured as GH binding to membranes of cells. Additionally, it was shown that insulin increased total GH binding in a concentration-dependent manner by up-regulating receptor biosynthesis^(36)^. Moreover, this former study showed that protein content of JAK2 was not affected by insulin^(36)^, which is in line with our results. In rats, it was shown that the hepatic GHR expression was reduced in the liver of diabetic animals, which could be inverted by insulin therapy^(37)^. In dairy cows, insulin treatment leads to increased GHR and IGF1 mRNA expression levels postpartum and therefore appeared to be a key metabolic signal in linking the GH–IGF1 axis^(24)^. Furthermore, it was shown that reduced serum insulin levels in mice led to lower GHR mRNA levels, indicating that the regulation of GHR expression by insulin is at the transcriptional level^(38)^. With regard to the results of these previous studies, it can be assumed that the modulation of GHR expression was regulated in an insulin-dependent manner in young goats, leading to a disruption of the somatotropic axis. Positive correlation between insulin and GHR expression levels supports this idea.

Hepatic INSR protein expression was significantly enhanced due to the protein-reduced diet, which might be a compensatory mechanism in response to reduced levels of insulin. As mRNA expression levels were unaltered, a translational modification of INSR was assumed. It is known that insulin is able to modulate the level of INSR. In the liver of GHR knock-out mice, the lack of GHR and GH was associated with increased INSR abundance, too^(39)^. It was presumed that this up-regulation was a result of lowered insulin concentrations observed in GHR knock-out mice^(39)^.

The decrease in insulin in the present study might be a result of diminished levels of blood Ca because a positive correlation between both parameters exists. Exocytosis of insulin in the pancreatic *β*-cells was controlled by Ca, and Ca channels are linked to insulin secretion^(40)^. It was described that Ca works as a regulator of insulin secretion, acting as a trigger for exocytosis^(41)^. Furthermore, insulin is a key signal of metabolic status^(24)^. As glucose concentrations did not differ between the two feeding groups, a decrease in insulin concentration due to reduced levels of blood Ca seems possible. Additionally, in rats fed a low-protein diet, the cAMP-dependent protein kinase that is important for insulin secretion in pancreatic *β*-cells was decreased^(42)^.

Increased protein expression levels of STAT5B and STAT3 might indicate compensatory mechanisms to maintain IGF1 synthesis. In rats fed a protein–energy malnutrition, IL-activated hepatic JAK2, STAT1 and STAT3 protein were enhanced^(43)^. In a study investigating impaired renal JAK-STAT signalling in chronic kidney disease, it was found that renal GHR expression was reduced and IL-6-mediated SOCS3 and STAT3 expression increased that may affect signalling along the GH-activated JAK-STAT pathway^(44)^. An increase in SOCS2 expression could contribute to diminished IGF1 levels. In line with the results of this previous study, renal GHR expression decreased in our study as well (data not shown). Though JAK2 was thought to be the most important GHR signalling kinase^(45)^, there is strong evidence that in murine pro-myeloid cell lines, activation of the GHR may result in an activation of Src family kinases, leading to activation of ERK1/2^(46)^ as an alternative pathway mediating GH activity. The Src is encoded by a proto-oncogene and activated by phosphorylation^(47)^. In mice with mutations in the JAK2-associated motif, it was shown that Src kinase could be activated by GHR independently of JAK2 and that a specific conformational change in the extracellular domain of GHR leads to the ERK1/2 signalling pathway instead of the JAK-STAT pathway^(46)^. Due to the protein-reduced diet, Src protein expression increased, whereas protein expression and phosphorylation of ERK1/2 remained unaltered. In human leukaemia cells, it was demonstrated that GH induced the activation of Src that in turn activated STAT5^(48)^. This might be an explanation for slightly enhanced protein expression levels of STAT5.

Studying the concept of feeding a protein-reduced diet to animals is of interest because in arid or semi-arid regions, feeding an adequate and biologically available protein amount to livestock might be difficult or even impossible due to inadequate substrates that are available in these climatic conditions.

From a broader point of view, the findings of the present study are of importance for humans as well because people in underdeveloped countries of the world might not be able to consume a sufficient amount of dietary protein or supply their children with the recommended amount of dietary protein every day, whereas low-priced carbohydrates are available. Moreover, the reduced intake of dietary protein might occur in the case of vegan cuisine. Here, people might not be aware of replacing animal proteins with plant proteins correctly and therefore enhancing their carbohydrate intake instead of consuming more protein to cover their energy demand.

Besides, for people suffering from kidney diseases, the findings of our study might be of relevance, as they might have to follow a strict low-protein diet for a long time or even for a lifetime. Especially for children who are growing, a reduced protein intake could affect the somatotropic axis through modulated GHR expression levels and therefore development and growth.

In summary, the results of the present study showed that feeding a protein-reduced diet to young goats modulated the somatotropic axis, leading to a reduction in IGF1. While the concentration of GH was not modified, the expression levels of hepatic GHR decreased due to reduced concentrations of insulin occurring in response to low levels of ionised Ca. As GHR is involved in many metabolic pathways, other parts of the body metabolism might be affected by such a diet. Further research is needed to clarify the influence of a protein-reduced diet on the feedback mechanism of the somatotropic axis, giving regard to the relation of reduced levels of IGF1 and hypothalamic hormones.
